# A pilot feasibility, safety and biological efficacy multicentre trial of therapeutic hypercapnia after cardiac arrest: study protocol for a randomized controlled trial

**DOI:** 10.1186/s13063-015-0676-3

**Published:** 2015-04-07

**Authors:** Glenn M Eastwood, Antoine G Schneider, Satoshi Suzuki, Michael Bailey, Rinaldo Bellomo, for the CCC trial investigators

**Affiliations:** Department of Intensive Care, Austin Hospital, Melbourne, Australia; School of Nursing and Midwifery, Deakin University, Melbourne, Australia; Australia New Zealand Intensive Care Society - Research Centre, Monash University, Melbourne, Australia; Department of Intensive Care Medicine and Burn Center, Centre Hospitalier Universitaire Vaudois, Lausanne, Switzerland; Department of Anesthesiology and Resuscitology, Okayama University Hospital, Okayama, Japan; Faculty of Medicine, University of Melbourne, Melbourne, Australia

**Keywords:** cardiac arrest, hypercapnia, intensive care, normocapnia, randomized trial, resuscitation

## Abstract

**Background:**

Cardiac arrest causes ischaemic brain injury. Arterial carbon dioxide tension (PaCO_2_) is a major determinant of cerebral blood flow. Thus, mild hypercapnia in the 24 h following cardiac arrest may increase cerebral blood flow and attenuate such injury. We describe the Carbon Control and Cardiac Arrest (CCC) trial.

**Methods/Design:**

The CCC trial is a pilot multicentre feasibility, safety and biological efficacy randomized controlled trial recruiting adult cardiac arrest patients admitted to the intensive care unit after return of spontaneous circulation. At admission, using concealed allocation, participants are randomized to 24 h of either normocapnia (PaCO_2_ 35 to 45 mmHg) or mild hypercapnia (PaCO_2_ 50 to 55 mmHg). Key feasibility outcomes are recruitment rate and protocol compliance rate. The primary biological efficacy and biological safety measures are the between-groups difference in serum neuron-specific enolase and S100b protein levels at 24 h, 48 h and 72 h. Secondary outcome measure include adverse events, in-hospital mortality, and neurological assessment at 6 months.

**Discussion:**

The trial commenced in December 2012 and, when completed, will provide clinical evidence as to whether targeting mild hypercapnia for 24 h following intensive care unit admission for cardiac arrest patients is feasible and safe and whether it results in decreased concentrations of neurological injury biomarkers compared with normocapnia. Trial results will also be used to determine whether a phase IIb study powered for survival at 90 days is feasible and justified.

**Trial registration:**

Australian New Zealand Clinical Trials Registry ACTRN12612000690853.

## Background

Cardiac arrest results in the immediate cessation of blood flow, sudden unconsciousness and, without prompt return of spontaneous circulation, irreversible brain injury within minutes [1,2]. Blood flow following return of spontaneous circulation, while vital to survival, also heralds the onset of cerebral reperfusion injury [3]. For cardiac arrest survivors, acute neurological injury exerts a profound effect on immediate, short-term and long-term quality of life outcomes [4,5]. Recent level I evidence has shown that the application of therapeutic hypothermia does not afford the previously accepted benefits of neurological protection or reduction in mortality [6]. Despite best efforts, the mortality outcomes for resuscitated cardiac arrest patients admitted to the intensive care unit remain high [7] and, in Australia and New Zealand, over the past decade intensive care unit mortality has essentially remained unchanged at 46% in 2003 and 48% in 2012 [8]. Thus, identifying modifiable aspects of post-resuscitation care to provide cerebral protection remains of extreme importance.

Arterial carbon dioxide tension (PaCO_2_) may be a modifiable component of patient care that might deliver improved neurological outcomes. Mild hypercapnia increases cerebral perfusion [9,10] is known to have anticonvulsant properties [11,12] as well as anti-inflammatory and anti-oxidant effects [13]. However, optimal PaCO_2_ levels during the early post-resuscitation period are currently poorly defined. Nonetheless, retrospective cohort studies have identified hypocapnia, both before admittance to [13,14] and within the intensive care unit [15-19], as being independently associated with worse neurological and mortality outcomes in cardiac arrest patients. Recently, a retrospective observational study of 16,542 patients enrolled at 125 participating intensive care units in Australia and New Zealand between 2000 and 2011 [18] found that, while hypercapnic patients had a similar mortality rate to normocapnic patients, they had a greater likelihood of being discharged home. Such findings were independently confirmed in a study of 409 out-of-hospital cardiac arrest patients from 21 Finnish intensive care units between March 2010 and February 2011 [11], and supported by observations on cardiac arrest in children [20] and experimental studies [21,22].

Therefore, mild hypercapnia during the 24 hours after cardiac arrest might contribute to positive neurological outcomes. Moreover, given that resuscitated cardiac arrest patients admitted to the intensive care unit typically receive mechanical ventilation, it should be feasible to target specific PaCO_2_ levels. This article describes a protocol for a pilot feasibility, safety and biological efficacy multicentre randomized controlled trial of therapeutic hypercapnia after cardiac arrest: the Carbon Control after Cardiac Arrest (CCC) trial.

## Methods/Design

The CCC trial is a prospective pilot, feasibility, safety and biological efficacy multicentre, randomized controlled trial.

Feasibility outcome measures include screened:recruited patient ratio, weekly recruitment rate, protocol adherence, time from cardiac arrest to first intensive care unit arterial blood gas analysis, separation in PaCO_2_ levels between groups, and ability to obtain Glasgow Outcome Scale (Extended) assessments at 6 months from the date of randomization for survivors. Safety outcome measures include adverse changes in serum concentrations of neuron-specific enolase and S100b protein (at 24 h, 48 h and 72 h), incidence and types of cardiac arrhythmia, adverse changes in acid-base values, oxygenation (mean arterial oxygen tension, fraction of inspired oxygen requirement, alveolar-arterial gradient, positive end expiratory pressure requirements) or in the findings of cardiac echocardiography or cerebral computed tomography during the first 24 h after admission to the intensive care unit, occurrence of cerebral edema or right ventricular failure, incidence of acute kidney injury as estimated using ‘risk, injury, failure, loss, end-stage’ renal disease (RIFLE) criteria, need for continuous renal replacement therapy, and liver failure.

The primary biological efficacy outcome measure is the difference in serum concentrations of neuron-specific enolase and S100b protein at 24 h, 48 h and 72 h after randomization between the hypercapnia and the control groups. Neuron-specific enolase (a cytoplasmic glycolytic enzyme) and S100b (a calcium binding protein) are biomarkers specific to the central nervous system [23,24]. Serum concentrations of neuron-specific enolase and S100b increase in both the cerebrospinal fluid and blood following a cardiac arrest. Both biomarkers are predictive of neurological outcome rather than survival outcome for resuscitated cardiac arrest patients [23,24]. Sustained elevated concentrations of neuron-specific enolase and S100b protein in the first 24 h to 72 h following cardiac arrest are associated with poor neurological outcome [24,25]. Secondary efficacy outcome measures include date, time and vital status at intensive care unit and hospital discharge and hospital discharge destination as clinical outcomes. A summary of the feasibility, safety and primary and secondary outcomes is provided in Table 1.

**Table 1:** **Primary and secondary outcomes of the CCC trial**

**Feasibility outcomes**	Screened:recruited patient ratio
	Weekly recruitment rate
	Time from cardiac arrest to first analysis of arterial blood gas in intensive care unit
	Separation in PaCO_2_ levels between groups for feasibility
	Protocol adherence
**Safety outcomes**	Adverse changes in cerebral injury biomarkers (at 24 h, 48 h, and 72 h)
	Incidence and type of cardiac arrhythmias
	Adverse changes in acid-base balance
	Adverse changes in oxygenation (mean arterial carbon dioxide tension, fraction of inspired oxygen, alveolar-arterial gradient, positive end expiratory pressure requirement)
	Adverse findings of cardiac echocardiography or cerebral computerized tomography
	Occurrence of cerebral oedema or right ventricular failure and incidence of acute kidney injury as estimated using RIFLE (‘risk, injury, failure, loss, end-stage’ renal disease) criteria, need for renal replacement therapy or liver failure
**Primary biological efficacy outcome**	Difference in serum neuron-specific enolase and S100b protein concentrations at 24 h, 48 h, and 72 h compared with baseline for each group
**Secondary outcomes**	Date, time and vital status at discharge from intensive care unit and hospital and discharge destination as clinical measures
	Glasgow Outcome Scale (Extended) assessed at 6 months from date of randomization for survivors, as a neurological assessment

### Study setting and participants

The intensive care units of four tertiary hospitals in Australia and New Zealand will participate in this trial. The CCC trial participants have to satisfy the protocol-defined inclusion and exclusion criteria.

### Inclusion criteria

- Resuscitated non-traumatic in-hospital or out-of-hospital cardiac arrest;
- Receiving mechanical ventilation;
- Admitted to intensive care unit.

### Exclusion criteria

- Spontaneous ventilation;
- Death considered imminent;
- Clinical or radiological suspicion of raised intracranial pressure or intracranial bleeding;
- Pregnant;
- Younger than 18 years;
- Severe chronic airflow limitation;
- Severe metabolic acidosis (pH < 7.1 and base excess < −6 mmol/l) uncorrected within the first 2 h of admission to intensive care unit;
- Transferred from another healthcare facility;
- Participation declined by the treating clinician.

### Randomization, allocation concealment and interventions

Randomization to either group will be performed using sealed opaque envelopes with permuted blocks of random sizes (block sizes 2 to 6) in a 1:1 ratio. Clinicians will be directed to target normocapnia (PaCO_2_ 35 to 45 mmHg) or mild hypercapnia (PaCO_2_ 50 to 55 mmHg) for 24 h during mechanical ventilation for each participant from the time of randomization. At all four sites, ventilation management, to adjust PaCO_2_ levels, will be achieved using intensive care unit-performed arterial blood gas analysis data assessed after adjustment to 37°C (alpha-stat) and guided on a minute-by-minute basis by end-tidal CO_2_ levels. After the 24 h intervention period, the treating clinician will determine the target PaCO_2_ level for each participant as per usual care. All remaining patient care decisions will be at the treating clinicians’ discretion. Blood samples will be centrifuged and isolated serum will immediately be frozen to −80°C at each participating site. Stored serum samples will then be shipped to the coordinating hospital and sent for batch analysis at a central laboratory in Melbourne, Victoria.

### Data collection and management

Trained research staff at each trial site will collect trial related data using a paper-based case report form. Information to be collected using the case report form is outlined in Table 2. Completed case report forms will be sent to the principal investigator at the Austin Hospital, and data will be collated and entered into a central trial database. The trial investigators at the Austin Hospital, Melbourne will develop and manage the trial database, coordinate sample shipment and batch analysis and will perform the data analysis.

**Table 2:** **Variables recorded in the CCC trial case report form**

**Baseline information**	Patient characteristics (age, sex, comorbities)
	Inclusion criteria and consent details
	Date and time of hospital admission
	Date and time of admission to intensive care unit
	APACHE II and III score on admission and immunocompromised status
	Date and time of randomization
**Cardiac arrest characteristics**	Location (in or out of- hospital), date and time of cardiac arrest
	Bystander cardiopulmonary resuscitation
	Time (minutes) until emergency responder arrival
	Initial cardiac rhythm
	Time of first defibrillation and number of defibrillation episodes
	Time of return of spontaneous circulation
	Total adrenaline dose
	Suspected cause of cardiac arrest (arrhythmic, hypoxic, ischaemic, pulseless electrical activity)
**Intensive care unit procedures and ventilation management**	Time of first arterial blood gas measurement and number of arterial blood gas measurements in first 24 h
	Therapeutic hypothermia initiated (yes or no)
	Nutrition commenced (yes or no)
	Coronary angiography in first 24 h
	Bicarbonate infusion commenced in first 24 h
	Recurrent cardiac arrhythmia (yes or no)
	Need for extracorporeal membrane oxygenation or renal replacement therapy
	Electroencephalography, computed tomography of the brain or echocardiography (yes and finding or not performed) in first 24 h
	Intravenous medication use (fentanyl, midazolam, neuromuscular blockage medication, midazolam, morphine, propofol)
	Duration of mechanical ventilation
	Arterial blood gas data during first 36 h of intensive care unit care while mechanically ventilated
	Ventilation data during first 36 h of intensive care unit care while mechanically ventilated
	Urinary and serum creatinine concentrations while in the intensive care unit
**Clinical outcomes**	Date, time and vital status at discharge from intensive care unit
	Date, time and vital status at discharge from hospital
	Destination after discharge from hospital
**Feasibility measures**	Screened:recruited patient ratio and weekly recruitment rate
	Separation in PaCO_2_ levels between groups
	Time (minutes) from cardiac arrest to first arterial blood gas analysis in intensive care unit
**Safety measures**	Occurrence of severe raised intracranial pressure or right ventricular failure, incidence of acute kidney injury as estimated using RIFLE (‘risk, injury, failure, loss, end-stage’ renal disease) criteria and liver failure
**Neurological evaluation measure**	Glasgow Outcome Scale (Extended) evaluation performed at 6 months

APACHE, Acute Physiology and Chronic Health Evaluation; PaCO_2_, arterial carbon dioxide tension.

### Ethical issues and trial registration

The Austin Hospital Human Research Ethics Committee is the lead ethics committee (approval: HREC/13/Austin/166; previously H2012/04737) and has provided approval for the Australian sites. Jurisdictional ethics committee approval has been obtained from the Health and Disability Ethics Committees, Ministry of Health, NZ (12/NTA/54) for the New Zealand site. Because of the specific nature of the trial, and the need to apply the intervention in a timely fashion, the need for prior informed consent was waived by the human research ethics committee. At the earliest appropriate time, the participant or their legal surrogate were asked for delayed consent. The trial was prospectively registered with the Australian New Zealand Clinical Trials Registry (ACTRN12612000690853).

### Statistical analysis

#### Trial profile

Flow of patients through the trial will be displayed in a Consolidated Standards of Reporting Trials (CONSORT) diagram. We will report the number of screened patients who met study inclusion criteria and the number of patients included in the study. We will report the reasons for exclusion of ineligible patients according to the predefined inclusion and exclusion criteria, as listed. In addition, we will report the number of patients enrolled into the trial and the number of participants from whom a full set of serum biomarker samples were obtained.

#### Statistical analysis procedures

The efficacy and safety of the intervention will be evaluated on an intention-to-treat analysis of all eligible participants randomized to the trial. A sample size of 50 participants with full serum biomarker samples (baseline, 24 h, 48 h and 72 h) was deemed sufficient to allow for a meaningful assessment of neurological biomarker difference between groups and for safety and feasibility outcomes. No imputation or assumptions will be made for missing data. Normally distributed continuous data will be reported as mean with standard deviation and compared using Student’s *t* test or analysis of variance (ANOVA). Non-normally distributed continuous data will be reported as median with interquartile range and compared using the Mann-Whitney U test or the Kruskal-Wallis one way ANOVA. Where sufficient symmetry exists, biomarker sample data for each participant for each time-point will be compared with repeated measures ANOVA; alternatively, non-parametric techniques will be employed. Categorical data will be reported as number and percentage and compared using chi-square or Fisher exact tests where indicated. Unless otherwise stated, a two-sided *P* value of 0.05 will be used to indicate statistical significance. We will also perform an adjusted analysis for key cardiac arrest or baseline characteristics or intensive care unit management imbalances. All analyses will be performed using SAS version 9.3 (SAS Institute Inc., Cary, NC, USA).

#### Tables and figures

Planned tables are:

- Baseline characteristics, cardiac arrest characteristics and intensive care unit procedures for the trial participants overall and according to study group.
- Primary and secondary outcome measures.

Planned figures are:

- CONSORT-style diagram illustrating the flow of patients through the trial.
- PaCO_2_ levels for normocapnia and mild hypercapnia group participants over the first 36 h following enrolment.
- Serum neuron-specific enolase concentrations for normocapnia and mild hypercapnia group participants with a complete set of neurological injury biomarkers (baseline, 24 h, 48 h, and 72 h).
- Serum S100b protein concentrations for normocapnia and mild hypercapnia group participants with a complete set of neurological injury biomarkers (baseline, 24 h, 48 h, and 72 h).

### Data and safety monitoring

No data monitoring committee was established for this investigator-initiated clinical trial. The principal investigator at each site will review safety data for the duration of the trial. All adverse events and serious adverse events linked with the study intervention will be reported to the Austin Health Human Research Ethics Committee within 24 h of study staff becoming aware of the event and in accordance with institutional and jurisdictional human research ethics committee requirements.

### Funding and support

The CCC trial is supported by the Australian and New Zealand Intensive Care Society Clinical Trials Group. Funding support has been received from the Anaesthesia Intensive Care Trust Fund (Austin Hospital, Melbourne), Intensive Care Foundation, Ambulance Victoria and the Austin Medical Research Foundation. Funding bodies have no input into the design, management or reporting of the trial.

## Discussion

The study commenced recruitment on 6 December 2012 at the Austin Hospital, Melbourne, Australia as single-centre trial. In October, 2013 the additional three participating sites joined the CCC trial, following the receipt of streamlined ethical approval, making this a multicentre trial. It is estimated that participant recruitment will be completed by November 2014 and final 6-month neurological assessment completed in May, 2015.

The results of the CCC trial will provide preliminary clinical evidence regarding the feasibility and safety of targeting mild hypercapnia for 24 h following intensive care unit admission for cardiac arrest patients. It will also provide preliminary information on whether such treatment leads to lower levels of neurological injury biomarkers concentrations, compared with normocapnia. Such trial results will be used to determine whether a phase IIb study is feasible, safe and justified.

## Trial status

Recruitment is active.

## Abbreviations

ANOVA: analysis of variance
APACHE: Acute Physiology and Chronic Health Evaluation
CCC: Carbon Control and Cardiac Arrest
CONSORT: Consolidated Standards of Reporting Trials
PaCO_2_: arterial carbon dioxide tension
RIFLE criteria: ‘risk, injury, failure, loss, end-stage’
